# Effects of *Launaea sarmentosa* Extract on Lipopolysaccharide-Induced Inflammation via Suppression of NF-κB/MAPK Signaling and Nrf2 Activation

**DOI:** 10.3390/nu12092586

**Published:** 2020-08-26

**Authors:** Thanh Q. C. Nguyen, Tran Duy Binh, Ryo Kusunoki, Tuan L. A. Pham, Yen D. H. Nguyen, Trong Tuan Nguyen, Kenji Kanaori, Kaeko Kamei

**Affiliations:** 1 Department of Functional Chemistry, Kyoto Institute of Technology, Kyoto 606-8585, Japan; nqcthanh@ctu.edu.vn (T.Q.C.N.); tdbinh22@gmail.com (T.D.B.); candyfloss.chemical@gmail.com (R.K.); phamleanhtuan.2807@gmail.com (T.L.A.P.); ndhyen94@gmail.com (Y.D.H.N.); kanaori@kit.ac.jp (K.K.); 2 Department of Chemistry, College of Natural Sciences, Can Tho University, Cantho City 94000, Vietnam; trongtuan@ctu.edu.vn

**Keywords:** *Launaea sarmentosa*, macrophages, inflammatory cytokine, antioxidant, endoplasmic reticulum stress, oxidative stress

## Abstract

*Launaea sarmentosa* has been extensively used as a nutrient herb in traditional Vietnamese remedies for the treatment of various diseases, especially inflammatory diseases. However, no detailed research has been conducted examining the molecular mechanisms involved in the suppression of inflammatory response. Here, we studied the effects of *L. sarmentosa* methanol extract on lipopolysaccharide (LPS)-induced inflammation using RAW 264.7 macrophages. The extract demonstrated potent antioxidant activity owing to the presence of polyphenolic and flavonoid components. Pretreatment with the extract inhibited LPS-mediated secretion of nitric oxide, reactive oxygen species, and tumor necrosis factor-α as well as the expression of inflammatory cytokines. Furthermore, the activation of the nuclear factor-kappa B pathway and phosphoinositide-3-kinase/protein kinase B pathways was blocked by the extract by inhibiting Akt phosphorylation. Additionally, the mitogen-activated protein kinase pathway was suppressed, and endoplasmic reticulum stress was attenuated. Furthermore, the extract promoted the activity of nuclear factor erythroid-2-related factor 2 resulting in the up-regulation of heme oxygenase-1 pathway, leading to the suppression of oxidative stress and inflammatory response. Taken together, the results indicate that *L. sarmentosa* exhibits anti-inflammatory effects, and hence, can be further developed as a novel drug for the treatment of diseases associated with excessive inflammation.

## 1. Introduction

Inflammation is described as a response to external noxious stimulation in fighting pathogens or endogenous damage signals, including cell damage to restore normal functions [1]. Hence, it is a critical biological process that plays an essential role in maintaining homeostasis in the human body. However, uncontrolled inflammatory processes lead to the overexpression of the pro-inflammatory mediators that are associated with several diseases such as diabetes, cardiovascular disease, atherosclerosis, heart failure, or cancer [2]. Administration of nonsteroidal and steroidal anti-inflammatory drugs is a common approach for the treatment of several inflammatory diseases. However, the adverse side effects involving hepatic and renal toxicities as well as cardiovascular events associated with such treatments is a cause of concern [3]. Therefore, the discovery of novel anti-inflammatory agents is a necessity for developing a treatment approach with minimum side effects associated with long-term use. Recently, traditional herbal medicine has garnered interest and is widely used as a complementary and alternative source for the treatment of inflammatory diseases [4]. Elucidating the molecular basis of the mechanism of action of herbal medicines can result in increased usage for the treatment of various diseases associated with chronic inflammation.

*Launaea sarmentosa* (Willd.) Schultz-Bip.ex Kuntze synonym *Launaea pinnatifida* (Cass.), a creeping herb, is extensively used in traditional Vietnamese remedies to treat many diseases, such as gout, urinary infection, and skin injuries. Based on Vietnamese remedies, *L. sarmentosa* is typically used as a nutritious vegetable or a supplement of mother’s milk after childbirth [5]. Previous phytochemical analyses of *L. sarmentosa* have confirmed the presence of alkaloids, carbohydrates, amino acids, steroids, glycosides, flavonoids, etc. Such compounds exhibit anti-inflammatory, antioxidant, and hepatoprotective activities [6]. Recently, it was reported that treatment with *L. sarmentosa* methanolic extract results in a reduction of the inflammatory response in the Carrageenan-induced hind paw enema rat model [7]. However, the mechanism of its anti-inflammation effects has not been fully elucidated.

Inflammatory stimuli, such as lipopolysaccharide (LPS), lead to the activation of macrophages. This stimulation initiates a biological response that results in the excessive production of inflammation mediators such as nitric oxide, cyclooxygenase, tumor necrosis factor-α [8]. Therefore, the expression of pro-inflammatory cytokines or inflammation mediators is used to evaluate the effects of pharmacological agents on inflammatory processes. During the inflammatory reaction stimulated by LPS, the activation of nuclear factor-kappa B (NF-κB) pathway and mitogen-activated protein kinase (MAPK) pathway can regulate the expression of pro-inflammatory cytokines and mediator factors [9]. In addition, oxidative stress resulting due to the inflammatory process is known to elevate the levels of intracellular ROS and regulate the expression of antioxidant enzymes as well as against oxidative damage to protect the tissues [10]. The regulation of genes encoding phase II detoxifying enzymes, especially heme oxygenase-1 (HO-1), is modulated by the nuclear translocation of the nuclear factor-erythroid-2-related factor (Nrf2) that plays a pivotal role in cell protection against oxidative stress and inflammation [11,12,13].

In this study, we evaluated the mechanism by which *L. sarmentosa* exerts its protective properties during LPS-induced inflammation in murine macrophage cell lines (RAW 264.7 macrophages). We demonstrated that *L. sarmentosa* methanol extract (Ls-ME) could attenuate the activation of the NF-κB/MAPK associated with the PI3K/Akt pathway and promote the Nrf2/HO-1 signaling pathway. Thus, we believe *L. sarmentosa* could be added to the pipeline of essential anti-inflammatory candidates to be developed as a potential treatment.

## 2. Materials and Methods

### 2.1. Chemicals and Reagents

Generally, all chemicals were obtained from FUJIFILM-Wako (Tokyo, Japan), unless stated otherwise. Fetal bovine serum (FBS) for cell culture was purchased from Gibco (GIBCO BRL, Grand Island, NY, USA). Cell Counting Kit-8 (CCK-8) and Cytotoxicity lactate dehydrogenase (LDH) Kit-WST were purchased from Dojindo Molecular Technologies (Rockville, MD, USA). Cellular ROS Kit (#ab113851) was obtained from Abcam (Tokyo, Japan). Nuclear Extraction (NE)-Kit and human TNF-alpha ELISA Ready-SET Go Kit (#88-7324) were obtained from eBiosciences (San Diego, CA, USA), respectively. Pierce bicinchoninic acid-(BCA) protein Kit and Enhanced chemiluminescent-(ECL) Western blotting substrate were purchased from Thermo Fisher Scientific (Waltham, MA, USA). Bovine serum albumin-(BSA) fraction V was purchased from Roche (Mannheim, Germany). All antibodies for Western blot analysis were purchased from Cell Signaling Technology (Tokyo, Japan), except anti-Nrf2 (Proteintech Corp., Tokyo, Japan), anti-p65 NF-ĸB, and horseradish peroxidase (HRP)-conjugated second antibody (Sigma-Aldrich, Burlington, MA, USA), anti-rabbit (IgG) Alexa Fluor^TM^ 488-conjugate (Alexa-488), anti-rabbit (IgG) Alexa Fluor^TM^ 594-conjugate (Alexa-594) (Molecular Probes, Invitrogen, CA, USA). DAPI (4′,6-diamino-2-phenylindole) dye used for the nuclei staining was purchased from Molecular Probes (Eugene, OR, USA).

### 2.2. Ls-ME Preparation

*L. sarmentosa* (voucher store: BT_La2019060003) was provided by the Department of Chemistry, Can Tho University, Vietnam. Briefly, the aerial parts of *L. sarmentosa* were dried naturally and pulverized to powder using a blender. Dried powder (10 g) was added to methanol (200 mL) at 25 °C for 1 day and supernatants were collected by centrifugation. The supernatants were filtered, concentrated using a vacuum evaporator (N-2100, EYELA, Tokyo, Japan) and subsequently freeze-dried. The extract was stored at −80 °C for further experiments. The amount of methanol extract obtained was 0.7 g. The concentrations of moisture and the ash of extract were evaluated as 14.30 ± 0.03% (*w/w*) and 16.93 ± 0.04% (*w/w*), respectively.

### 2.3. Radical Scavenging Activity

The effect of Ls-ME on 2,2-diphenyl-1-picrylhydrazyl (DPPH) reagent was evaluated based on the protocol established in a previous study with minor modifications [14]. Briefly, 0.96 mL various concentrations of Ls-ME (0, 10, 20, 30, 40, 50, and 60 µg/mL) were added to 0.04 mL DPPH (1 mg/mL). Finally, the absorbance was determined using a spectrophotometer at 517 nm (V-730 UV-Vis; Jasco, Tokyo, Japan). The results were compared with Vitamin C, which was used as a positive control.

### 2.4. Total Polyphenolic, Flavonoid and Polysaccharide Content

The total polyphenolic content of Ls-ME was determined using the modified Folin–Ciocalteu’s assay [15]. An equal volume of the plant extract in methanol (50 µg/mL) and the Folin–Ciocalteu reagent was mixed and added to 0.2 mL sodium carbonate solution (10%; *w/v*). The sample was mixed with a vortex and incubated for 30 min at 40 °C. The absorbance was determined at 765 nm. The result was compared with Gallic acid, which was used as control. Total polyphenolic content was determined as mg/g gallic acid equivalent (GAE) of dried extract.

The total flavonoid content of Ls-ME was analyzed, following the aluminum chloride (AlCl_3_) method with a minor modification [16]. An aliquot of 0.25 mL plant extract was added to 0.15 mL AlCl_3_ (10%; *w/v*), 0.15 mL potassium acetate (1 M), and 0.2 mL distilled water. The samples were incubated for 30 min at room temperature, followed by the measurement of absorbance at 415 nm. Quercetin was used as a standard. Total flavonoid content was evaluated as mg/g quercetin equivalence (QE) of dried extract.

The total polysaccharide content of Ls-ME was determined using the phenol-sulfuric acid method established by a previous study with minor modifications. An equal volume of the plant extract and phenol 5% reagent was mixed, followed by the addition of 5 mL of concentrated sulfuric acid. The samples were incubated for 10 min, and the absorbance was determined at 488 nm [17,18]. Total polysaccharide content was evaluated as the percentage of polysaccharide in the dried extract.

### 2.5. Cell Culture and Cell Viability Assay

Murine macrophage cell line RAW 264.7 (ATCC^®^ TIB-71^TM^, Manassas, VA, USA) was cultured in Dulbecco’s modified Eagle’s medium (DMEM) containing FBS (10%; *v/v*) and PS (1%; *v/v*), at 37 °C in a humidified incubator with 5% CO_2_.

Ls-ME (1 g/mL) was dissolved in DMSO (5%; *v/v*) and used as the stock. The stock was prepared with the appropriate sample concentration before use. While administering Ls-ME treatment to the cells, the final concentration of DMSO was maintained at 0.5% (*v/v*).

Cell viability based on the measurement of intracellular dehydrogenase activity was evaluated by CCK-8 assay. Briefly, cells were cultured and seeded in 96 well-plates (2 × 10^5^ cells/well) and treated with a range of Ls-ME concentrations (0, 50, 100, 200, 400, and 800 µg/mL) for 24 h following incubation with or without LPS (1 µg/mL) for 18 h. The absorbance was determined at 450 nm using a microplate reader (SH-1200, Ibaraki, Japan).

### 2.6. Cell Cytotoxicity Assay

To assess cellular damage, LDH Kit-WST was used to measure lactate dehydrogenase (LDH) activity in the medium according to the manufacturer’s instruction. Whole-cell lysates were prepared using 2% (*v/v*) Triton X-100.

### 2.7. Nitric Oxide and Reactive Oxygen Species Production Assay

The quantification of nitric oxide (NO) production was performed using Griess reagent according to the protocol established in a previous study [19]. The absorbance was determined at 540 nm, and the standard curve was calculated using sodium nitrite to determine NO concentration [20]. L-NAME (100 μM) was used as an inhibitor of inducible nitric oxide synthase (iNOS; positive control).

Cellular ROS assay Kit was used to measure ROS production based on the conversion of the fluorescence from 2′,7′-dichlorodihydrofluorescein diacetate (DCFH2–DA) according to the manufacturer’s instruction. Fluorescence corresponding to the intracellular ROS level was measured at 488/535 nm using a spectrofluorometer (Wallac 1420; Perkin-Elmer, Turku, Finland).

### 2.8. TNF-a Production

The levels of TNF-α protein were analyzed using the ELISA kit according to the manufacturer’s instructions. The standard curve derived using the TNF-α mouse standard was used to determine the concentration of TNF-α. After pretreatment with various concentrations of Ls-ME for 24 h, cells were incubated with LPS (1 µg/mL) for 18 h. The TNF-α protein was quantified using a colorimetric reaction based on activity of avidin-horseradish peroxidase (bound to the biotinylated detection antibody) on a specific substrate. The supernatants were stored at −80 °C for further experiments.

### 2.9. Reverse Transcription–Quantitative PCR (RT-qPCR)

Cells (2 × 10^6^ cells/well) were cultured in six-well plates for 24 h. The cells were then treated with Ls-ME for 24 h, washed with PBS, and stimulated with or without LPS (1 µg/mL) for 18 h. The Qiazol reagent was used to extract total RNA, which was subsequently purified using the Qiagen RNeasy kit following the manufacturer’s instruction. Total RNA was stored at −80 °C until further analysis. The effects of Ls-ME on the mRNA expression were analyzed by RT-qPCR as described in a previous report [19]. The following primers were used: *iNOS* forward: 5′-GGAGCCTTTAGACCTCAACAGA-3′ reverse: 5′-AAGGTGAGCTGAACGAGGAG-3′; interleukin-6 (*IL-6*) forward: 5′-GCTACCAAACTGGATATAATCAGGA-3′ reverse: 5′-CCAGGTAGCTATGGTACTCCAGAA-3′; interleukin-1β (*IL-1β*) forward: 5′-AGTTGACGGACCCCAAAAG-3′ reverse: 5′-AGCTGGATGCTCTCATCAGG-3′; cyclooxygenase-2 (*COX-2*) forward: 5′-GATGCTCTTCCGAGCTGTG-3′ reverse: 5′-GGATTGGAACAGCAAGGATTT-3′; C/EBP homologous protein (*CHOP*) forward: 5′-GATGCACTTCCTTCTGGAACA-3′ reverse: 5′-GCGACAGAGCCAGAATAACA-3′; X-box binding protein 1 (*XBP-1*) 5′-TGGGCATCTCAAACCTGCTT-3′ reverse: 5′-GCGTCCAGCAGGCAAGA-3′; *β-Actin* forward: 5′-GGAGGGGGTTGAGGTG-3′ reverse: 5′-GTGTGCACTTTTATTGGTCTCA-3′. PCR amplification efficiency was determined for each gene by serial dilutions of target cDNA, described in Supplementary Table S1.

### 2.10. Western Blotting

Cells (2 × 10^6^ cells/well) were cultured and seeded on a 6-well plate. After overnight preincubation, cells were incubated with a selected concentration of Ls-ME for 24 h following LPS-stimulation (1 µg/mL) for 18 h. Whole proteins were extracted from each sample using RIPA lysis buffer. NE kit was used to extract cytoplasmic and nuclear proteins, and the protein concentrations were analyzed using the BCA protein kit. A total of 20 µg protein of each sample was separated using 10% (*v/v*) sodium dodecyl-sulfate polyacrylamide gel electrophoresis (SDS-PAGE). The samples were subsequently transferred onto polyvinylidene fluoride-(PDVF) membranes. After blocking with skim milk (5%; *w/v)* in tris-buffered saline Tween-20 (TBST) (0.5%; *v/v)* for 60 min, the membrane was incubated with primary antibodies and phosphorylated (p-) antibodies (p-IĸB*α*, p-p38 I, p-ERK½, p-JNK, p-Akt, IκB*α*, p65 NF-κB, p38, ERK½, JNK, Akt, HO-1, lamin-B1, Nrf2, β-actin) overnight at 4 °C according to the manufacturer’s recommendation. Both cytosol and nuclear protein fractions were used to analyze p65 NF-κB and Nrf2. The nuclear protein fraction was used to analyze lamin B1 (nuclear loading control), and the whole-cell extract containing total proteins was used to analyze other proteins. After incubation, the membranes were washed with TBST at least 5 times (5 min), then incubated with HRP-conjugated secondary antibody in skim milk (5%; *w/v*) or BSA (3%; *w/v*) for 1 h at room temperature. Finally, the membranes were washed at least 5 times (5 min) in TBST and visualized using ECL Western blotting Substrate. The band of proteins was detected using AE-9300H Ez-Capture MG (ATTO Corp., Tokyo, Japan). The Image J analysis software (Nation Institute of Health, Sacaton, AZ, USA) was used to quantify the band intensity. These band intensities were normalized to β-Actin and lamin-B1, corresponding to cytosolic proteins and nucleic proteins, respectively.

### 2.11. Immunofluorescence Staining

Cells (2 × 10^4^ cells/well) were cultured in eight-well chamber slides (Thermo Fisher Scientific, Nunc Lab-Tek Chamber Slides, Waltham, MA, USA), followed by treatment with a selected concentration of Ls-ME with or without LPS for 30 min in accordance with the protocol described in previous studies [21]. The cells were incubated either with rabbit anti-p65 NF-κB (1:800) followed by Alexa-488 (1:1000) or rabbit anti-Nrf2 (1:800) followed by Alexa-594 (1:800). The nuclei were stained with DAPI for 20 min. Samples were washed and mounted on a glass slide, followed by inspection with a fluorescence scanning microscope FV10i (Olympus, Tokyo, Japan). The MetaMorph analysis software (Molecular Devices, Sunnyvale, CA, USA) was used to analyze intensities.

### 2.12. Statistical Analysis

All experiments were performed in triplicates at the least to confirm reproducibility, and all data are expressed as means ± SD. Statistical analyses were performed using *t*-tests and one-way ANOVA, and *p*-values of <0.05 were considered statistically significant.

## 3. Results

### 3.1. Antioxidant Activity of Ls-ME

The antioxidant activity of the methanol extract of *L. sarmentosa* (Ls-ME) was examined by evaluating its scavenging effect on DPPH radical (data are not shown). The free radical-scavenging activity enhanced linearly with increasing concentrations of Ls-ME. The SC_50_ value of Ls-ME, corresponding to the concentration required to scavenge 50 % of the free radicals in the sample, was observed to be 26.7 μg/mL (equivalent to 5.5 μg/mL vitamin C). In addition, the total amounts of polyphenols and flavonoids were observed to be 258.2 ± 0.78 mg GAE/g of dried extract and 71.2 ± 0.37 mg QE/g of dried extract, respectively. Additionally, the total polysaccharide content in Ls-ME was observed to be 0.11 % (*w/w*). These results demonstrated that Ls-ME could scavenge free radicals effectively.

### 3.2. Effects of Ls-ME on the Viability of LPS-Stimulated RAW 264.7 Macrophages 

**Figure 1:**
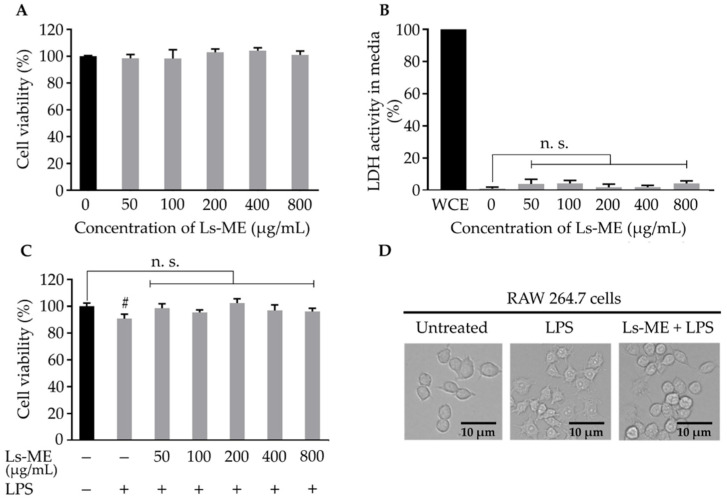
The effects of Ls-ME on the viability and morphology of LPS-stimulated RAW 264.7 cells. Cells were treated with various concentrations of Ls-ME for 24 h. (**A**) Cell viability and (**B**) LDH activity were examined. LDH activity in whole-cell extract (WCE) is represented as 100% activity and used as the control. The effects of Ls-ME on cell viability after LPS stimulation are demonstrated in (**C**). The morphology of cells was examined in three groups: the untreated group with medium only, group treated with LPS (LPS-1 µg/mL) for 18 h, and Ls-ME + LPS pretreatment group (Ls-ME 200 μg/mL for 24 h) (LPS-stimulation was performed for 18 h). The morphology of the cells is shown in (**D**). The results are represented as mean ± SD (n = 6). Scale bar indicates 10 µm in (**D**); ^#^ *p* < 0.05; n. s., not significant in comparison with the untreated group.

Before the anti-inflammatory assay, the cytotoxicity of Ls-ME on RAW 264.7 macrophages was assessed by examining cell survival in the presence or absence of LPS. This step was performed to find an optimal concentration of Ls-ME that exhibits minimum toxicity. No significant effect was observed on cell survival upon treatment with Ls-ME with concentrations up to 800 µg/mL (Figure 1A). Typically, the amount of LDH released from dead cells was an indicator used to evaluate the cytotoxicity. The LDH levels did not increase in the medium upon treatment with Ls-ME (Figure 1B). These results suggest that Ls-ME does not exert cytotoxic activity with concentrations up to 800 µg/mL.

During LPS stimulation, cell viability was reduced, as reported previously. The effect of pretreatment with Ls-ME followed by LPS stimulation was also investigated. As expected, cell viability was reduced significantly upon stimulation with LPS than that in the non-treated group. Pretreatment with Ls-ME suppressed the reduction in cell viability (Figure 1C). With respect to the morphology of activated macrophages, the LPS-treated group demonstrated an altered shape in comparison with the untreated group [22]. The cells retained original round shape in the group that was subjected to pretreatment with Ls-ME despite stimulation with LPS (Figure 1D). These results demonstrated that Ls-ME does not exhibit cytotoxicity and protects cells from LPS-induced damage.

### 3.3. Ls-ME Reduces NO, ROS, and TNF-α Production

**Figure 2:**
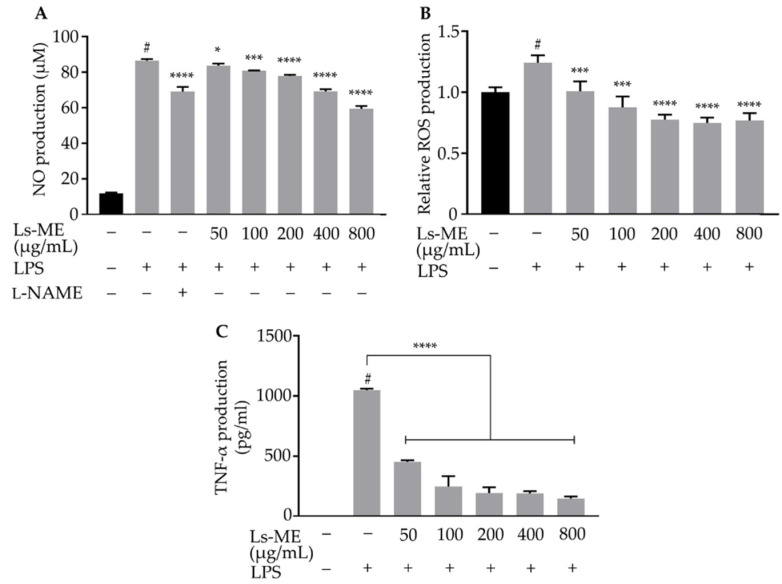
The suppression effects of Ls-ME on LPS-induced NO, ROS and TNF-α production in RAW 264.7 macrophages. Cells were cultured with serial concentrations of Ls-ME for 24 h, followed by treatment with LPS (1 µg/mL) for 18 h. The NO levels in LPS-stimulated cells were measured using the Griess assay (**A**). The NO level observed upon pretreatment with L-NAME at the concentration of 100 µM was used as a positive control. Intracellular ROS accumulation was evaluated (**B**). TNF-α production in LPS-treated cells was determined using ELISA (**C**). Data obtained from three replicates are represented as mean ± SD. ^#^ *p* < 0.05 is expressed in comparison with cells incubated alone; * *p* < 0.05; *** *p* < 0.001 and **** *p* < 0.0001 are expressed in comparison with LPS treatment only.

Since NO and ROS are well-known pro-inflammatory mediators that play an essential role in the inflammatory process, the effects of Ls-ME on LPS-induced inflammatory response in RAW 264.7 cells were investigated by the detection NO levels and intracellular ROS production [22,23]. The generation of NO was remarkably enhanced in response to the stimulation with LPS. However, pretreatment with Ls-ME significantly suppressed the production of NO (Figure 2A). Furthermore, the ROS production was up-regulated upon stimulation with LPS, while significantly downregulated upon pretreatment with Ls-ME (Figure 2B).

Additionally, the levels of pro-inflammatory cytokine as TNF-α were analyzed using ELISA. An increase in TNF-α production was demonstrated in LPS-stimulated macrophages, while it was significantly suppressed upon pretreatment with Ls-ME (Figure 2C). Collectively, these findings indicate that Ls-ME inhibits the production of inflammatory mediators and cytokine in LPS-stimulated RAW 264.7 cells. Based on these results, 50–200 µg/mL concentrations of Ls-ME were selected for subsequent experiments to further evaluate the mechanism underlying its anti-inflammatory effects.

### 3.4. Ls-ME Suppressed the Expression of Pro-Inflammatory Genes

**Figure 3:**
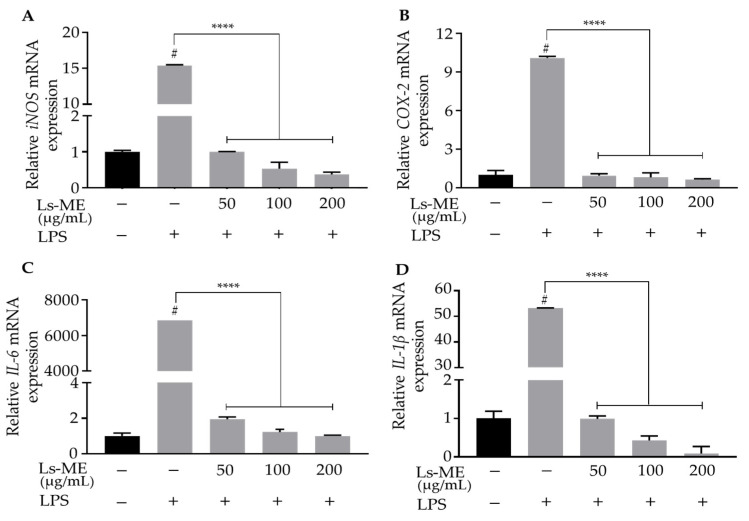
The inhibitory activity of Ls-ME on LPS-induced mRNA expression corresponding to pro-inflammatory genes in RAW 264.7 macrophages. Cells were cultured with serial concentrations of Ls-ME for 24 h, followed by treatment with LPS (1 µg/mL) for 18 h. The mRNA levels of *iNOS* (**A**), *COX-2* (**B**), *IL-6* (**C**), and *IL-1β* (**D**) genes were determined by RT-qPCR. Data of the three replicates are represented as mean ± SD. ^#^ *p* < 0.05 is expressed in comparison with cells incubated alone; **** *p* < 0.0001 is expressed in comparison with LPS treatment only; n. s., not significant.

For the examination of the protective role of Ls-ME, we next examined the transcriptional levels of *iNOS* (*NOS2*), *COX-2*, *IL-6*, and *IL-1β* by RT-qPCR analysis. As expected, the mRNA expression of these pro-inflammatory genes was up-regulated upon LPS stimulation than those in the control group, whereas pretreatment with Ls-ME significantly down-regulated the increasing mRNA levels of the aforementioned cytokines (Figure 3). Our results suggest that Ls-ME suppresses the mRNA levels of pro-inflammatory genes, thereby mitigating the inflammation.

### 3.5. Ls-ME Attenuates the Activation of NF-ĸB/PI3K/Akt Pathways

**Figure 4:**
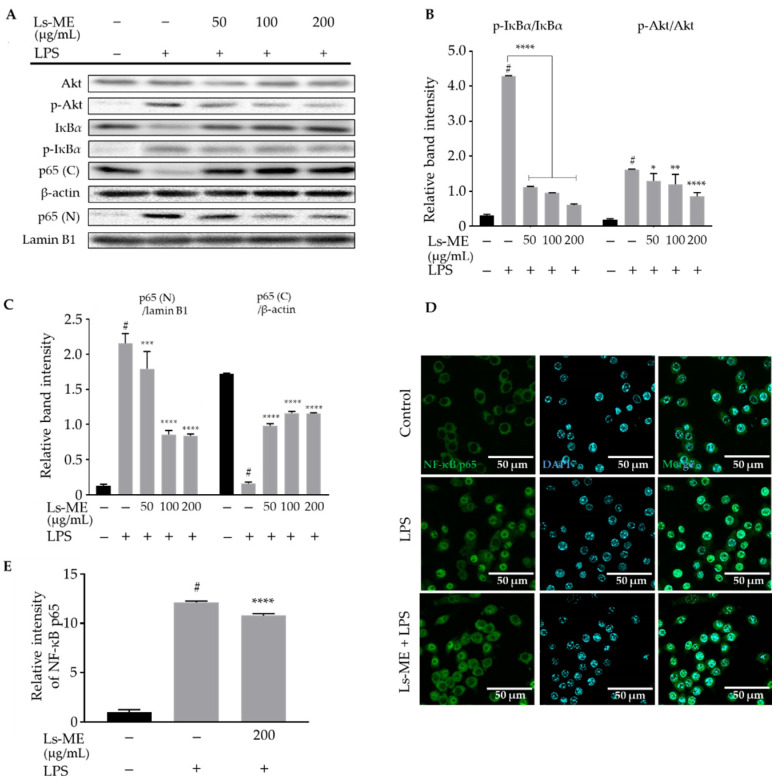
The inhibitory activity of Ls-ME on the activation of NF-κB/PI3K/Akt pathways in LPS-stimulated RAW 264.7 macrophages. Cells were cultured with serial concentrations of Ls-ME for 24 h, followed by treatment with LPS (1 µg/mL) for 18 h. Western blot analysis determined the expression of total Akt, IκB*α* and phosphorylated p-Akt, p-IĸB*α* (**A**). Cytosol and nuclear fractions were separated in whole-cell lysates to analyze p65 (C, cytosol) and p65 (N, nucleus). The relative band intensity of p-IκBα to IκBα and that of p-Akt to Akt was quantified (**B**). The relative band intensity of p65 (C, cytosol) and p65 (N, nucleus) was quantified (**C**). The relative band intensities were analyzed using Image J. Representative images are shown in (**D**) with the green color of Alexa-488. Control, cells incubated alone; LPS, cells incubated with LPS; Ls-ME + LPS, cells incubated with Ls-ME (200 µg/mL), then treated with LPS. The fluorescent intensities were analyzed by MetaMorph (*n* = 35) (**E**). Scale bar indicates 50 µm. Data of three replicates are represented as mean ± SD. ^#^ *p* < 0.05 is expressed in comparison with cells incubated alone; * *p* < 0.05; ** *p* < 0.01; *** *p* < 0.001; **** *p* < 0.0001 are expressed in comparison with LPS treatment only.

NF-κB and its upstream regulator PI3K/Akt signaling pathway are activated in the inflammatory process and promote the expression of inflammatory mediators [23]. Based on the aforementioned results, we further explored whether the effects of Ls-ME on LPS-induced inflammation were observed as a result of its influence on NF-κB and PI3K/Akt signaling pathways using Western blot analysis. As demonstrated in Figure 4A,B, an increase in the expression level of p-Akt and p-IκBα was suppressed significantly upon pretreatment with Ls-ME.

In addition, LPS enhanced the nuclear translocation of the p65 subunit. However, the levels of p65 subunit in the nucleus significantly reduced upon Ls-ME treatment (50–200 µg/mL) before the LPS stimulation (Figure 4A,C). Furthermore, the NF-κB p65 subunit was visualized using immunofluorescence staining to confirm its localization under the influence of Ls-ME. Consistent with these findings, the results imply that LPS-triggered localization of the p65 subunit was attenuated upon treatment with Ls-ME (Figure 4D,E). Hence, our results suggest that Ls-ME possesses the ability to suppress NF-κB/PI3K/Akt activation.

### 3.6. Ls-ME Suppresses the Activation of MAPKs Signaling Pathway

**Figure 5:**
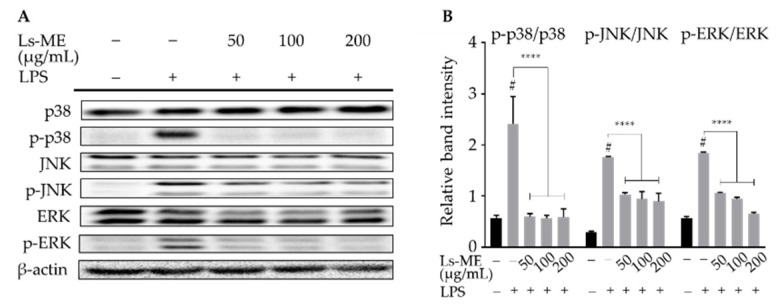
The inhibitory activity of Ls-ME on LPS-induced phosphorylation of MAPKs signaling pathway in RAW 264.7 macrophages. Cells were cultured with serial concentrations of Ls-ME for 24 h, followed by treatment with LPS (1 µg/mL) for 18 h. The expression of total p38, JNK, ERK½, and phosphorylated p-p38, p-JNK, p-ERK½ were examined using Western blot analysis (**A**). Relative band intensities were analyzed using Image J (**B**). Data of the three replicates are represented as mean ± SD. ^#^ *p* < 0.05 is expressed in comparison with cells incubated alone; **** *p* < 0.0001 is expressed in comparison with LPS treatment only.

During the inflammatory process, the activation of MAPKs family, including extracellular signal-regulated kinases ½ (ERK½), c-Jun amino (N)-terminal kinases (JNK), and p38 MAPK, also plays an essential modulatory role in the adaption and initiation of the transcription of inflammation mediators [24]. Western blot analyses demonstrated that LPS stimulation activated the phosphorylation of ERK½, JNK, and p38 MAPK, whereas their phosphorylation was effectively suppressed upon pretreatment with Ls-ME (Figure 5). The results indicate that Ls-ME can mediate anti-inflammatory activity due to its inhibitory effects on the phosphorylation of MAPKs.

### 3.7. Ls-ME Suppresses ER-Stress

**Figure 6:**
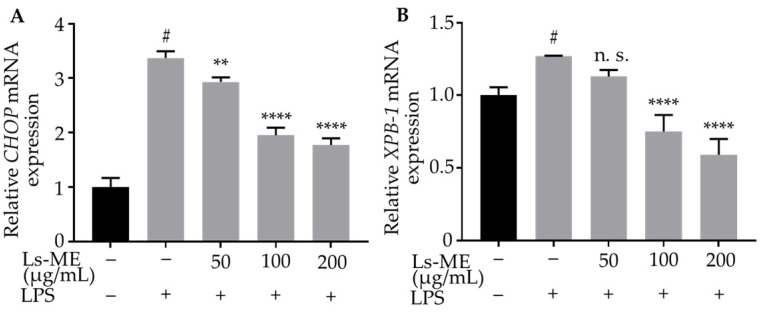
Ls-ME suppressed the expression of *CHOP* (**A**) and *XBP-1* (**B**) in LPS-stimulated RAW 264.7 cells. Cells were cultured with serial concentrations of Ls-ME for 24 h, followed by treatment with LPS (1 µg/mL) for 18 h. The mRNA levels of *CHOP*, *XBP-1* were analyzed using RT-qPCR. Data of the three replicates are represented as mean ± SD. ^#^ *p* < 0.05 is expressed in comparison with cells incubated alone; ** *p* < 0.01; **** *p* < 0.0001 are expressed in comparison with LPS treatment only; n. s., not significant.

Endoplasmic reticulum (ER)-stress is a potential factor that can aggravate the inflammatory response, and the C/EBP homologous protein (CHOP) is one of the critical markers for determining the effects of ER-stress [25]. Additionally, the depletion of X-box-binding protein1 (XBP-1) mRNA, which is a vital component of ER-stress, indicates significant inhibition of the expression of inflammatory mediators [26]. To evaluate the effect of Ls-ME on ER stress, we measured the mRNA levels of *CHOP* and *XBP-1* using RT-qPCR. As illustrated in Figure 6, the mRNA levels of *CHOP*, *XBP-1* were enhanced in response to LPS stimulation. However, the levels were significantly reduced upon pretreatment with Ls-ME. These results demonstrate that Ls-ME reduces ER-stress that is induced by stimulation with LPS in RAW 264.7 macrophages.

### 3.8. Ls-ME Promotes the Nrf2/HO-1 Activation

**Figure 7:**
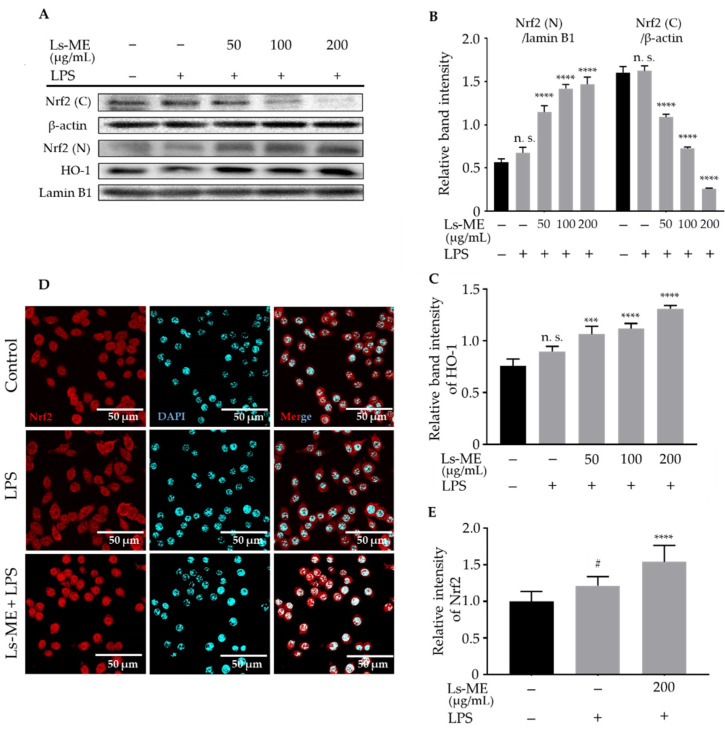
Ls-ME promoted the Nrf2/HO-1 activation. Cells were cultured with serial concentrations of Ls-ME for 24 h, followed by treatment with LPS (1 µg/mL) for 18 h. The expression of Nrf2 in the cytosol (C) and nucleus (N) fractions, and HO-1 in the whole-cell lysate was determined using Western blot (**A**). Relative band intensities of Nrf2/ β-actin, Nrf2 (N)/lamin B1 (**B**), and HO-1/lamin B1 (**C**) were quantified using Image J. The cells were cultured in medium only (control), LPS only (1 µg/mL), or Ls-ME (200 µg/mL) followed by LPS (1 µg/mL) and were stained with the Nrf2 antibody labeled with Alex Fluor 594 (red) and DAPI (blue). Representative images are shown in (**D**) with the red color of Alexa-594. Control, cells incubated alone; LPS, cells incubated with LPS; Ls-ME + LPS, cells incubated with Ls-ME (200 µg/mL), then treated with LPS. The fluorescent intensities were analyzed by MetaMorph (*n* = 35) (**E**). Scale bar indicates 50 µm. Data of the three replicates are represented as mean ± SD. ^#^ *p* < 0.05 is expressed in comparison with cells incubated alone; *** *p* < 0.001; **** *p* < 0.0001 is expressed in comparison with LPS treatment only; n. s., not significant.

Nrf2-mediated expression of genes encoding antioxidants reduces ROS production and contributes to the anti-inflammatory effects [27]. We further evaluated whether the Nrf2/HO-1 pathway is associated with the antioxidant effects of Ls-ME. First, the results of the Western blot with nuclear proteins indicated that Ls-ME promoted Nrf2 nuclear translocation and effectively up-regulated HO-1 level (Figure 7A–C). Moreover, immunostaining showed enhanced localization of Nrf2 in the nucleus (Figure 7D,E). Taken together, these findings demonstrated that Ls-ME exerts its anti-inflammatory activity by inducing the Nrf2/HO-1 activation.

## 4. Discussion

Numerous herbal remedies have been documented with distinct clinical benefits for the overall health and/or the prevention of inflammatory diseases. Remarkably, epidemiological evidence suggests that traditional medicinal herbs that are potential sources of novel anti-inflammatory therapeutics exert bioactivities with fewer toxic side effects [28,29]. *L. sarmentosa* was previously reported to exert pharmacological effects, indicating its potential in the treatment for various diseases, especially inflammation [6]. Nevertheless, an examination of the detailed and comprehensive mechanism of action behind its pharmacological properties and chemical constituents is still required. We observed that Ls-ME consists of a high amount of polyphenol (258.2 ± 0.78 mg GAE/g of dried extract); however, relatively small amounts of flavonoids (71.2 ± 0.37 mg QE/g of dried extract) and polysaccharides (0.11%) were observed, which is consistent with previous studies. These results can be attributed to the fact that most of the polyphenolic components in the extract are non-flavonoid compounds [5]. Polyphenols flavonoids and polysaccharides are the primary contributors to the anti-oxidative process [30,31]. Ls-ME demonstrated a high DPPH scavenging activity (SC_50_: 26.7 µg/mL). This result indicates that the anti-oxidative property can be attributed to the high polyphenolic content. The antioxidant properties of *L. sarmentosa* are relatively more potent than the antioxidant properties of other plant extracts [5,32]. A recent study observed that *Anacyclus clavatus* is a potential anti-inflammatory herb with lower levels of phenolics and flavonoids than those in *L. sarmentosa* [33]. Collectively, these results indicated that *L. sarmentosa* should be further evaluated at a scientific level and for its potential in therapeutic applications.

Antioxidants (polyphenol and flavonoid) can scavenge free radicals, thereby suppressing the oxidative damage to the cellular structures. Therefore, antioxidants have been explored as active agents to protect cells against inflammation [34]. In particular, the role of the oxidative stress caused by LPS has been demonstrated during the examination of inflammatory processes, especially during the initiation or progression period [35]. As shown in Figure 1C,D, the results of the evaluation of cell viability and the morphological observation indicated that Ls-ME protects the cells from LPS-induced cell damage. Furthermore, our results demonstrated that Ls-ME significantly inhibited the essential inflammatory mediators in LPS stimulation, such as NO, ROS, TNF-α production (Figure 2). In response to typical stimulants such as LPS after infection, immune cells secrete NO and other products in response to inflammation. Excessive levels of NO may lead to inflammatory diseases [36]. The overexpression of iNOS is a major cause of excessive NO secretion. In this investigation, Ls-ME significantly inhibited the NO secretion and the expression of iNOS, demonstrating an effective anti-inflammatory response (Figure 3A). Additionally, immune cells also release prostaglandin E2 (PGE_2_), which is produced by COX-2 in the inflammatory process [37]. As expected, Ls-Me effectively inhibited the expression of COX-2 (Figure 3B). Therefore, these results indicate that Ls-ME may relieve some symptoms of inflammation. Besides, the expression of pro-inflammation cytokines (IL-6, IL-1β) is enhanced during an immune reaction. Ls-ME significantly down-regulated the increased expression of IL-6 and IL-1β (Figure 3C,D). IL-6 is a soluble mediator with a pleiotropic effect on the inflammatory response, immune response, and hematopoiesis [38]. The elevated expression of IL-1β has been observed in various human cancers [39]. Based on these findings, further research should elucidate the molecular mechanism of the anti-inflammatory activity of *L. sarmentosa* to develop new medicines.

LPS activates the innate immune system by binding to Toll-like receptor-4 (TLR-4), which leads to the activation of a signaling cascade, including the NF-κB pathway. Once the NF-κB is activated, the translocation of NF-κB subunits is enhanced, leading to a pro-inflammatory response [35]. In unstimulated cells, NF-κB exists as a heterodimer composed of p50/p65 subunits and is sequestered in the cytosol by specific IκBs [40]. LPS can activate the phosphorylation of IκBα leading to its degradation, allowing the NF-κB to translocate into the nucleus where it activates the transcription of genes [41,42]. Our results demonstrated that Ls-ME significantly suppresses the IκBα phosphorylation, resulting in the inhibition of the localization of the p65 NF-κB subunit upon stimulation with LPS in RAW 264.7 macrophages (Figure 4). Pro-inflammatory cytokines also induce the expression of free radicals such as NO, ROS. Additionally, excessive free radicals can cause oxidative stress; hence, free radicals can both repress or activate signaling cascades [43,44]. Therefore, the reduction of the expression of free radicals such as NO, ROS by Ls-ME may affect NF-κB activation.

NF-κB activation is promoted by a variety of cellular protein kinases, especially MAPKs (p38, ERK, JNK) and PI3K/Akt, which play vital roles in the inflammatory process [35]. A previous study demonstrated that the activation of Akt in LPS-stimulated cells acts as an upstream regulator of NF-κB in microglial cells [45]. Additionally, a recent study reported that the stimulation of LPS might lead to the phosphorylation of MAPK in macrophages [46]. Akt was first represented by its function in the regulation of cell differentiation and proliferation [47]. The present study indicated that Ls-ME inhibits Akt phosphorylation (Figure 4A,B) and MAPKs activation induced by LPS (Figure 5).

Several cellular events mediated by LPS stimulation may trigger the activation of certain signaling pathways that lead to the production of ROS and ER stress [48]. To evaluate the possible activation of pathways, the production of ER-stress was examined by analyzing the expression levels of CHOP and XBP-1 that act as ER-stress markers related to the pathogenesis in inflammation [25,49,50]. A previous study demonstrated that p38 MAPK activated the expression of CHOP in stressed cells [51]. Interestingly, Endo et al. showed that ER-stress was triggered by LPS stimulation, followed by the overexpression of CHOP in ER-stress response. It also triggers p38 activation, which mediates the process of apoptosis [49]. Our study demonstrated that the expressions of CHOP and XBP-1 were enhanced upon LPS-stimulation, whereas the effects of LPS were significantly suppressed by Ls-ME. Collectively, these findings reinforce our hypothesis that *L. sarmentosa* exerts its anti-inflammatory effect by blocking NF-κB/MAPK/PI3K/Akt signaling pathways to attenuate ER-stress induced in RAW 264.7 macrophages upon stimulation by LPS.

Oxidative stress is observed during LPS stimulation, leading to the activation of macrophages and a potent inflammatory reaction [52]. Therefore, we evaluated the anti-oxidative stress effects of Ls-ME in LPS-stimulated macrophages. A previous study demonstrated that Nrf2 activation and antioxidant signaling pathway can prevent the upregulation of transcription of genes, such as IL-6, which is induced by LPS. Moreover, Nrf2 activation reduces ROS production by mediating the expression of genes encoding antioxidants [27]. Our results showed that Ls-ME significantly inhibited the increase in cellular damage, ROS production, and expression levels of IL-6 induced by LPS (Figure 1D, Figure 2B and Figure 3C), suggesting that Ls-ME can exert anti-oxidative stress and anti-inflammatory effects by promoting the activity of Nrf2 pathway. Nrf2 appears in the cytoplasm as it binds to Kelch-like ECH-associated protein (Keap1) under normal conditions. However, its translocation can exhibit effects on oxidative stress response and up-regulate the level of anti-oxidative enzymes, especially HO-1 [53]. We observed that pretreatment with Ls-ME enhanced the nuclear translocation of Nrf2 upon LPS stimulation (Figure 7). We believe that enhancing Nrf2-dependent response may be a potential target for preventing inflammation and oxidative stress. HO-1 is one of the effective mediators associated with the inflammatory response that depends on the activation of Nrf2 [54]. Our study demonstrated that the level of HO-1 was enhanced by Ls-ME (Figure 7C). These findings illustrate that Ls-ME can promote the activity of the Nrf2/HO-1 signaling pathway to up-regulate the HO-1 levels, thereby reducing ROS production. These findings are consistent with the results of our previous study indicating that *Lasia spinosa* leaf extract suppresses the inflammation induced by LPS-stimulation via the activation of the Nrf2/HO-1 [19]. Polyphenols and flavonoids can affect inflammation and antioxidant pathways by regulating the transcriptional factors such as NF-κB, Nrf2 [55,56]. Additionally, the presence of other components such as polysaccharides, terpenes, and terpenoids in Ls-ME may also affect its biological activity [57,58]. Therefore, Nrf2/HO-1 activation may also be regulated by these components.

In conclusion, we proved that Ls-ME suppresses the inflammatory mediators by inhibiting the NF-κB/MAPK/PI3K/Akt pathway and promoting the activation of Nrf2/HO-1 signaling pathway in LPS-stimulated RAW 264.7 macrophages. Additionally, Ls-ME can attenuate ER-stress, resulting in decreased levels of CHOP and XBP-1. Our findings provide the initial evidence of the mechanism underlying the anti-inflammatory action of *L. sarmentosa* and highlight its potential as a phytotherapeutic agent. Further research is needed to isolate active compounds and evaluate its effects on inflammation.

## Supplementary Materials

The following are available online at https://www.mdpi.com/2072-6643/12/9/2586/s1, Supplementary Table S1: RT-qPCR amplification efficiency for proinflammatory genes. PCR amplification efficiency was determined for each gene by serial dilutions of target cDNA.

## Abbreviations

LPS	Lipopolysaccharide
PS	Penicillin-streptomycin
DMSO	Dimethyl sulfoxide
MAPK	Mitogen-activated protein kinase
IκBα	Nuclear factor of kappa light polypeptide gene enhancer in B-cells inhibitor, alpha
PI3K	Phosphoinositide-3-kinase/protein kinase B
RIPA	Radioimmunoprecipitation

## References

[1] Fürst R., Zündorf I. (2014). Plant-derived anti-inflammatory compounds: Hopes and disappointments regarding the translation of preclinical knowledge into clinical progress. Mediat. Inflamm..

[2] Scrivo R., Vasile M., Bartosiewicz I., Valesini G. (2011). Inflammation as “common soil” of the multifactorial diseases. Autoimmun. Rev..

[3] Aminoshariae A., Kulild J.C., Donaldson M. (2016). Short-term use of nonsteroidal anti-inflammatory drugs and adverse effects: An updated systematic review. J. Am. Dent. Assoc..

[4] Ghasemian M., Owlia S., Owlia M.B. (2016). Review of Anti-Inflammatory Herbal Medicines. Adv. Pharmacol. Sci..

[5] Them L.T., Tuong Nguyen Dung P., Thi Nhat Trinh P., Tong Hung Q., Tuong L.N., Trong Tuan N., Duc Lam T., Thuy Nguyen V., Dung L.T. (2019). Saponin, Polyphenol, Flavonoid content and α-glucosidase Inhibitory Activity, Antioxidant Potential of *Launaea sarmentosa* Leaves grown in Ben Tre province, Vietnam. IOP Conf. Ser. Mater. Sci. Eng..

[6] Makwana H.T., Pandya D.J. (2019). *Launaea pinnatifida* Cass. A Species of the Controversial Drug Gojihva: Comprehensive Review. Int. J. Pharmacogn. Phytochem. Res..

[7] Raju G.S., Moghal M.M.R., Hossain M.S., Hassan M.M., Billah M.M., Ahamed S.K., Rana S.M.M. (2014). Assessment of pharmacological activities of two medicinal plant of Bangladesh: *Launaea sarmentosa* and *Aegialitis rotundifolia* roxb in the management of pain, pyrexia and inflammation. Biol. Res..

[8] Ji K.Y., Kim K.M., Kim Y.H., Im A.R., Lee J.Y., Park B., Na M.K., Chae S. (2019). The enhancing immune response and anti-inflammatory effects of *Anemarrhena asphodeloides* extract in RAW 264.7 cells. Phytomedicine.

[9] Choi Y.H., Kim G.Y., Lee H.H. (2014). Anti-inflammatory effects of cordycepin in lipopolysaccharide-stimulated RAW 264.7 macrophages through Toll-like receptor 4-mediated suppression of mitogen-activated protein kinases and NF-κB signaling pathways. Drug Des. Dev. Ther..

[10] Kwon D.H., Cha H.J., Choi E.O., Leem S.H., Kim G.Y., Moon S.K., Chang Y.C., Yun S.J., Hwang H.J., Kim B.W. (2018). Schisandrin A suppresses lipopolysaccharide-induced inflammation and oxidative stress in RAW 264.7 macrophages by suppressing the NF-κB, MAPKs and PI3K/Akt pathways and activating Nrf2/HO-1 signaling. Int. J. Mol. Med..

[11] Huang Y., Li W., Su Z.Y., Kong A.N.T. (2015). The complexity of the Nrf2 pathway: Beyond the antioxidant response. J. Nutr. Biochem..

[12] Loboda A., Damulewicz M., Pyza E., Jozkowicz A., Dulak J. (2016). Role of Nrf2/HO-1 system in development, oxidative stress response and diseases: An evolutionarily conserved mechanism. Cell. Mol. Life Sci..

[13] Kang K.A., Hyun J.W. (2017). Oxidative stress, Nrf2, and epigenetic modification contribute to anticancer drug resistance. Toxicol. Res..

[14] Tailor Chandra Shekhar G.A. (2014). Antioxidant Activity by DPPH Radical Scavenging Method of *Ageratum conyzoides*. Am. J. Ethnomed..

[15] Singleton V., Othofer R., Lamuela-Raventos R.M. (1999). Analysis of Total Phenols and Other Oxidation Substrates and Antioxidants by Means of Folin-Ciocalteu Reagent. Methods Enzymol..

[16] Aryal S., Baniya M.K., Danekhu K., Kunwar P., Gurung R., Koirala N. (2019). Total Phenolic content, Flavonoid content and antioxidant potential of wild vegetables from western Nepal. Plants.

[17] He J., Chen L., Chu B., Zhang C. (2018). Determination of total polysaccharides and total flavonoids in *chrysanthemum morifolium* using near-infrared hyperspectral imaging and multivariate analysis. Molecules.

[18] Jiang B., Zhang H., Liu C., Wang Y., Fan S. (2010). Extraction of water-soluble polysaccharide and the antioxidant activity from *Ginkgo biloba* leaves. Med. Chem. Res..

[19] Nguyen T.Q., Duy Binh T., Pham T.L., Nguyen Y.D., Dai Trang T.X., Nguyen T.T., Kanaori K., Kamei K. (2020). Anti-Inflammatory Effects of *Lasia spinosa* Leaf Extract in Lipopolysaccharide-Induced RAW 264.7 Macrophages. Int. J. Mol. Sci..

[20] Schmölz L., Wallert M., Lorkowski S. (2017). Optimized incubation regime for nitric oxide measurements in murine macrophages using the Griess assay. J. Immunol. Methods.

[21] Chen R. (2012). Immunoflurescence (Indirect Staining) Protocol for Adherent Cells. Bio-Protocol.

[22] Dai B., Wei D., Zheng N.N., Chi Z.H., Xin N., Ma T.X., Zheng L.Y., Sumi R., Sun L. (2019). Coccomyxa gloeobotrydiformis polysaccharide inhibits lipopolysaccharide-induced inflammation in RAW 264.7 macrophages. Cell. Physiol. Biochem..

[23] Jin Y., Liu L., Chen B., Bai Y., Zhang F., Li Q., Lv C., Sun H., Li J., Rubby S. (2017). Involvement of the PI3K/Akt/NF-κB Signaling Pathway in the Attenuation of Severe Acute Pancreatitis-Associated Acute Lung Injury by *Sedum sarmentosum* Bunge Extract. BioMed Res. Int..

[24] Jeong Y.E., Lee M.Y. (2018). Anti-inflammatory activity of *populus deltoides* leaf extract via modulating NF-κB and p38/JNK pathways. Int. J. Mol. Sci..

[25] Zhang K., Kaufman R.J. (2008). From endoplasmic-reticulum stress to the inflammatory response. Nature.

[26] Gargalovic P.S., Gharavi N.M., Clark M.J., Pagnon J., Yang W.P., He A., Truong A., Baruch-Oren T., Berliner J.A., Kirchgessner T.G. (2006). The unfolded protein response is an important regulator of inflammatory genes in endothelial cells. Arterioscler. Thromb. Vasc. Biol..

[27] Kobayashi E.H., Suzuki T., Funayama R., Nagashima T., Hayashi M., Sekine H., Tanaka N., Moriguchi T., Motohashi H., Nakayama K. (2016). Nrf2 suppresses macrophage inflammatory response by blocking proinflammatory cytokine transcription. Nat. Commun..

[28] Yuan H., Ma Q., Ye L., Piao G. (2016). The traditional medicine and modern medicine from natural products. Molecules.

[29] Beg S., Swain S., Hasan H., Barkat M.A., Hussain M.S. (2011). Systematic review of herbals as potential anti-inflammatory agents: Recent advances, current clinical status and future perspectives. Pharmacogn. Rev..

[30] Dzoyem J.P., Eloff J.N. (2015). Anti-inflammatory, anticholinesterase and antioxidant activity of leaf extracts of twelve plants used traditionally to alleviate pain and inflammation in South Africa. J. Ethnopharmacol..

[31] Wang J., Hu S., Nie S., Yu Q., Xie M. (2016). Reviews on Mechanisms of in Vitro Antioxidant Activity of Polysaccharides. Oxid. Med. Cell. Longev..

[32] Azam S., Ansari P., Rashid M.M.U., Alam M.N., Ahmed I.H., Ibarahim M.Y., Shafi S.M., Rahman S., Hossen A. (2015). In vitro anti-oxidant and in vivo anti-inflammatory activity determination of the methanolic leaves extract of *Millettiapachycarpa*. Biomed. Res. Ther..

[33] Bouriche H., Kherbache A., Kada S., Senator A., Demirtas I. (2016). Phenolic content, anti-inflammatory and antioxidant activities of *Anacyclus clavatus* extracts. Environ. Exp. Biol..

[34] Lin D., Xiao M., Zhao J., Li Z., Xing B., Li X., Kong M., Li L., Zhang Q., Liu Y. (2016). An overview of plant phenolic compounds and their importance in human nutrition and management of type 2 diabetes. Molecules.

[35] Wu H., Wang Y., Zhang Y., Xu F., Chen J., Duan L., Zhang T., Wang J., Zhang F. (2020). Breaking the vicious loop between inflammation, oxidative stress and coagulation, a novel anti-thrombus insight of nattokinase by inhibiting LPS-induced inflammation and oxidative stress. Redox Biol..

[36] Bogdan C., Röllinghoff M., Diefenbach A. (2000). Reactive oxygen and reactive nitrogen intermediates in innate and specific immunity. Curr. Opin. Immunol..

[37] Leite P.C., Nunes C., Jamal S.K., Cuccovia I.M., Salette R. (2017). Nonsteroidal Anti-Inflammatory Therapy: A Journey Toward Safety. Med. Res. Rev..

[38] Tanaka T., Narazaki M., Kishimoto T. (2014). IL-6 in Inflammation, Immunity, and Disease. Cold Spring Harb. Perspect. Biol..

[39] Baker K.J., Houston A., Brint E. (2019). IL-1 family members in cancer; two sides to every story. Front. Immunol..

[40] Liang Y., Zhou Y., Shen P. (2004). NF-κB and Its Regulation on the Immune System. Cell. Mol. Immunol..

[41] Liu T., Zhang L., Joo D., Sun S.C. (2017). NF-κB signaling in inflammation. Signal Transduct. Target. Ther..

[42] Tak P.P., Firestein G.S. (2001). NF-κB: A key role in inflammatory diseases. J. Clin. Investig..

[43] Lingappan K. (2018). NF-κB in Oxidative Stress. Curr Opin. Toxicol..

[44] Akanda M.R., Kim I.S., Ahn D., Tae H.J., Nam H.H., Choo B.K., Kim K., Park B.Y. (2018). Anti-inflammatory and gastroprotective roles of *rabdosia inflexa* through downregulation of pro-inflammatory cytokines and MAPK/NF-κB signaling pathways. Int. J. Mol. Sci..

[45] Saponaro C., Cianciulli A., Calvello R., Dragone T., Iacobazzi F., Panaro M.A. (2012). The PI3K/Akt pathway is required for LPS activation of microglial cells. Immunopharmacol. Immunotoxicol..

[46] Han J.M., Lee E.K., Gong S.Y., Sohng J.K., Kang Y.J., Jung H.J. (2019). Sparassis crispa exerts anti-inflammatory activity via suppression of TLR-mediated NF-κB and MAPK signaling pathways in LPS-induced RAW264.7 macrophage cells. J. Ethnopharmacol..

[47] Cantley L.C. (2002). The phosphoinositide 3-kinase pathway. Science.

[48] Pereira D.M., Correia-da-Silva G., Valentão P., Teixeira N., Andrade P.B. (2014). Anti-inflammatory effect of unsaturated fatty acids and ergosta-7,22-dien-3-ol from Marthasterias glacialis: Prevention of CHOP-mediated ER-stress and NF-κB activation. PLoS ONE.

[49] Endo M., Mori M., Akira S., Gotoh T. (2006). C/EBP Homologous Protein (CHOP) is Crucial for the Induction of Caspase-11 and the Pathogenesis of lipopolysacchride-induced inflammation. J. Immunol..

[50] Nakayama Y., Endo M., Tsukano H., Mori M., Oike Y., Gotoh T. (2010). Molecular mechanisms of the LPS-induced non-apoptotic ER stress-CHOP pathway. J. Biochem..

[51] Wang X.Z., Ron D. (1996). Stress-induced phosphorylation and activation of the transcription factor CHOP (GADD153) by p38 MAP kinase. Science.

[52] Zuo L., Prather E.R., Stetskiv M., Garrison D.E., Meade J.R., Peace T.I., Zhou T. (2019). Inflammaging and oxidative stress in human diseases: From molecular mechanisms to novel treatments. Int. J. Mol. Sci..

[53] Ren J., Li L., Wang Y., Zhai J., Chen G., Hu K. (2019). Gambogic acid induces heme oxygenase-1 through Nrf2 signaling pathway and inhibits NF-κB and MAPK activation to reduce inflammation in LPS-activated RAW264.7 cells. Biomed. Pharmacother..

[54] Park S.Y., Park D.J., Kim Y.H., Kim Y.H., Kim S.G., Shon K.J., Choi Y.W., Lee S.J. (2011). Upregulation of heme oxygenase-1 via PI3K/Akt and Nrf-2 signaling pathways mediates the anti-inflammatory activity of Schisandrin in Porphyromonas gingivalis LPS-stimulated macrophages. Immunol. Lett..

[55] Yahfoufi N., Alsadi N., Jambi M., Matar C. (2018). The immunomodulatory and anti-inflammatory role of polyphenols. Nutrients.

[56] Leyva-López N., Gutierrez-Grijalva E.P., Ambriz-Perez D.L., Basilio Heredia J. (2016). Flavonoids as cytokine modulators: A possible therapy for inflammation-related diseases. Int. J. Mol. Sci..

[57] Hou C., Chen L., Yang L., Ji X. (2020). An insight into anti-inflammatory effects of natural polysaccharides. Int. J. Biol. Macromol..

[58] Gonzalez-Burgos E., Gomez-Serranillos M.P. (2012). Terpene Compounds in Nature: A Review of Their Potential Antioxidant Activity. Curr. Med. Chem..

