# Supplementary material for: Effects of Launaea sarmentosa Extract on Lipopolysaccharide-Induced Inflammation via Suppression of NF-κB/MAPK Signaling and Nrf2 Activation
Source: Nutrients. 2020 Aug 26;12(9):2586. doi: 10.3390/nu12092586 (PMC7551017; doi:10.3390/nu12092586)
Supplement: Supplementary file 1 [file nutrients-12-02586-s001.pdf]

Supplementary Table: RT-qPCR amplification efficiency for proinflammatory genes. PCR amplification efficiency was determined for each gene by serial dilutions of target cDNA, described in the table.

Gene	Slope	R^2	Amplification efficiency (%)
iNOS	-3.318	0.9972	100.16
COX-2	-3.2643	0.9922	102.46
IL-6	-3.2633	0.9948	102.51
IL-1β	-3.2471	0.9963	102.12
CHOP	-3.2017	0.9961	101.35
XBP-1	-3.3514	0.9760	98.87
β-actin	-3.2112	0.9931	104.37

Primer's name; inducible nitric oxide synthase (iNOS), cyclooxygenase-2 (COX-2), interleukin-6 (IL-6), interleukin-1β (IL-1β), C/EBP homologous protein (*CHOP*), X-box binding protein 1 (*XBP-1*), β-Actin.
